# Laser-Induced Molecular Plasma: A Medium for High-Order Harmonics Generation of Ultrashort Pulses

**DOI:** 10.3390/ijms23147613

**Published:** 2022-07-09

**Authors:** Rashid A. Ganeev

**Affiliations:** 1 Laboratory of Nonlinear Optics, University of Latvia, Jelgavas 3, LV-1004 Riga, Latvia; rashid.ganeev@lu.lv; 2 Tashkent Institute of Irrigation and Agricultural Mechanization Engineers, National Research University, Kori Niyozov Str. 39, Tashkent 100000, Uzbekistan; 3 Department of Physics, Voronezh State University, 394006 Voronezh, Russia

**Keywords:** molecular plasma, high-order harmonics generation, optimization of plasma formation

## Abstract

I present a study of laser-induced plasmas (LIPs) produced on the surfaces of molecular targets to create optimal conditions for high-order harmonics generation during the propagation of femtosecond pulses through the LIP. The resonance enhancement of a harmonic, two-color pump of plasma, quasi-phase-matching and nanoparticle-induced growth of the harmonic yield are analyzed, which allows for the formation of sources of coherent extreme ultraviolet radiation based on molecular plasma formation.

## 1. Introduction

High-order harmonics generation (HHG) is a way of generating coherent extreme ultraviolet (XUV) radiation. Usually, gaseous media [1] and specular reflection from surfaces [2] are used for HHG. Among gaseous media, noble atomic gases are the most frequently used species for this process [3], though a few molecular gases have also allowed for the generation of high-order harmonics at a lesser conversion efficiency [4,5].

The formation of atomic and molecular media during the laser ablation of solid materials offers another approach for the application of such materials for HHG. Laser-induced plasma (LIP) has demonstrated its attractiveness as an advanced medium for harmonics generation. Numerous atomic targets have been used for these purposes [6,7,8,9,10,11,12,13,14,15,16,17,18,19,20,21]. Various advanced processes, such as the resonance enhancement of a single harmonic, two-color pump of plasma, quasi-phase-matching and nanoparticle-induced growth of harmonic yield, have been reported, allowing for the formation of sources of coherent XUV radiation. Molecular targets, though applied from time to time for these purposes, have demonstrated less attractive features of harmonics enhancement compared with atomic targets [9,10,20,22,23]. The reasons for the lesser efficiency of HHG in molecular LIPs were related to some restricting factors, which did not allow for the application of the above-mentioned advanced processes for the increase in the harmonic yield.

In this paper, I demonstrate the optimization of molecular LIP for HHG formation. I show the conditions of molecular target ablation when resonance-induced processes start to play a decisive role in the enhancement of harmonics. I also demonstrate the quasi-phase-matching of a group of harmonics generated in molecular LIPs. Finally, I present perspectives on the enhancement of a whole set of harmonics when femtosecond pulses propagate through nanoparticles (NPs) comprising molecular structures. 

## 2. Results and Discussion

### 2.1. Resonance-Induced Amplification of a Single Harmonic

**Figure 1:**
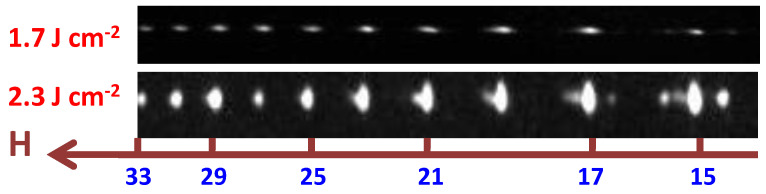
Harmonic spectra from Cr_3_C_2_ LIP in the case of 806 nm driving pulses and different fluencies of heating pulses. “H” refers to the harmonic order. Upper panel: HHG in chromium carbide plasma at weak ablation (F = 1.7 J cm^−2^). Bottom panel: HHG in chromium carbide plasma at stronger ablation (F = 2.3 J cm^−2^). In the latter case, H29 was enhanced with regard to H27.

First, I studied the possibility of the amplification of a single harmonic in the region of the harmonic distribution plateau. In the case of the single-color pump (SCP) of Cr_3_C_2_ plasma, no single-harmonic amplification was observed when I used a relatively weak heating pulses (HP) fluence (F = 1.7 J/cm^2^) on the surface of the target. A featureless, gradually fading spectrum of odd harmonics was generated (see the raw image of the harmonic spectrum shown in the upper panel of Figure 1). Note that the atomic chromium target often showed the resonance-induced amplification of a single harmonic in a 28 nm vicinity. An increase in the fluence to 2.3 J/cm^2^ led to the appearance of an amplified 29th harmonic (H29, lower panel in Figure 1).

Among different atomic species, chromium metal became an attractive object of HHG studies (for example, [24]), since it allowed for the observation of different effects of harmonic enhancements (quasi-phase-matching of harmonics in the vicinity of strong autoionizing states and harmonic enhancement due to the formation of laser-induced small-sized agglomerates). The determination of the conditions of ablation at which the above processes could be realized, simultaneously, seemed as an attractive challenge. The present HHG study in molecular chromium plasma was performed at an 80 ns delay between the HP and driving pulses (DP). Notice that an approximately equal harmonic yield was achieved along the 60–90 ns range of delay variations. This study showed that the time-resolved emission spectrum from the laser ablation was a clear indicator, allowing for the determination of optimum conditions for HHG in the chromium-contained plasma.

The most specific feature of the HHG spectrum from this plasma was the appearance of enhanced H29 (*λ* = 27.8 nm) of 800 nm class lasers, which were notably stronger than the lower-order harmonics [24]. This unusual distribution of harmonics in the plateau-like region of the XUV was attributed to the resonance-induced enhancement of this harmonic in the vicinity of strong ionic transitions possessing strong oscillator strength (*gf*). Previous studies of the photoabsorption and photoionization spectra of atomic Cr plasma in the range of 41–42 eV [25] have demonstrated the presence of strong transitions, which could be responsible for the suppressed pattern of the harmonic spectrum in the wavelength region of 29.5–31 nm. This effect could be seen in the case of the suppressed H27 (*λ* = 29.9 nm) in the bottom panel of Figure 1. At the same time, the region of the 3*p* → 3*d* transitions (27.6–28.2 nm, *gf* = 0.63) of Cr II spectra was analyzed in [26]. The authors showed that some of these transitions could enhance the nonlinear optical response of the plasma. The present study of molecular Cr-contained plasma showed that the proper excitation of the ablating target plays a crucial role in the formation of the conditions when the resonance-induced process starts playing an important role in the modification of the harmonic distribution, from a featureless, gradually decaying pattern of harmonics to a spectrum containing strong harmonics in the shorter wavelength range (Figure 1).

I would like to emphasize that the purpose of this study was to qualitatively determine the effect of molecular plasma on the efficiency of harmonic conversion under various plasma formation conditions. The raw images shown in Figure 1 and some other figures were presented for a better viewing of the harmonics distribution. Quantitative measurements of harmonic spectra in plasma were presented in some other figures.

### 2.2. HHG in Multiparticle Molecular Structures

The comparative study of HHG in plasma consisting of either molecular NP or monomers showed that, under optimal experimental conditions, the former emitters provided a higher harmonic output, which indicated the improved properties of harmonics in the plasmas containing molecular NPs. Here, I present the study of molecular NP-contained plasmas (In_2_O_3_ and Mn_2_O_3_), allowing for the efficient generation of lower-order harmonics and the resonance enhancement of a single (13th) harmonic (in the case of In_2_O_3_ NP LIP).

The sizes of Mn_2_O_3_ NPs were in the range of 40–60 nm. In_2_O_3_ NPs were in the range of 20–70 nm. The morphology of the NPs before and after ablation was analyzed using a transmission electron microscope. An analysis of the material being removed showed NPs similar to the original ones. This observation confirmed the presence of molecular NPs in the plasma plume at the HP flow rates used. NP targets were prepared by mixing with glue. Note that the glue that ablated separately from the NPs did not allow for the generation of high-order harmonics.

**Figure 2:**
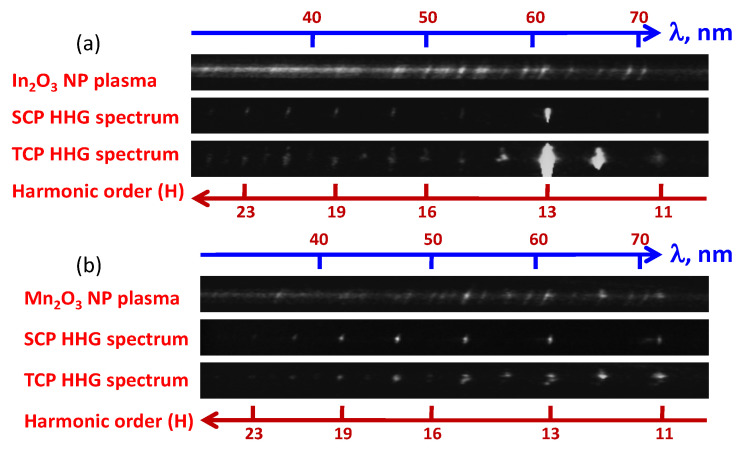
(**a**) Spectra of plasma (upper panel) and harmonics (SCP in the case of the middle panel and two-color pump (TCP) in the case of the bottom panel) produced from In_2_O_3_ NP plasma (F = 0.7 J cm^−2^ in the case of the two bottom panels and F = 1.3 J cm^−2^ in the case of the upper panel). (**b**) Spectra of plasma and harmonics (upper panel) and harmonics without plasma emission (two bottom panels) during ablation of Mn_2_O_3_ NPs. The two bottom panels were obtained using F = 0.8 J cm^−2^ and the upper panel was obtained at 1.5 J cm^−2^. Pure harmonic spectra were obtained in the case of SCP (806 nm, middle panel) and TCP (806 nm and 403 nm, bottom panel).

Only an incoherent emission of plasma was observed during the ablation of In_2_O_3_ nanoparticles at the fluence F = 1.3 J cm^−2^. At these conditions, a large number of free electrons prevented the phase-matching of harmonics (upper panel of Figure 2a). Meanwhile, at the smaller fluence (0.7 J cm^−2^), plasma emission disappeared and the strong harmonics dominated in the observed XUV spectrum (middle panel of Figure 2a). The characteristic feature of this spectrum was the enhanced single harmonic (H13) of 806 nm radiation. Harmonics up to H27 were observed in the case of SCP.

In the case of the two-color pump (TCP, 806 nm + 403 nm), strong H10 and H12 were observed, alongside H13 (Figure 2a, bottom panel). The collection time of the CCD camera determining harmonic spectra was increased by a factor of three compared with the case shown in the middle panel of Figure 2a to demonstrate a difference between the enhanced odd harmonic (H13) and other odd and even harmonics. Overall, the TCP of this LIP led to the enhancement of H12.

The upper panel of Figure 2b shows the short-wavelength plasma emission and weak low-order harmonics up to H15 generated in the plasma produced on the manganese oxide target at the fluence F = 1.5 J cm^−2^. A decrease in the fluence allowed for the exclusion of the plasma emission and the influence of the free electrons on the conversion efficiency of harmonics. Stronger low-order harmonics were observed in the case of Mn_2_O_3_ NP-contained plasma at the optimal conditions of the excitation of the NP-covered target (F = 0.8 J cm^−2^, Figure 2b, middle panel). The two-color pump of Mn_2_O_3_ NP LIP allowed for the generation of some relatively strong odd and a few even harmonics (H12 and H14) using this plasma (Figure 2b, bottom panel). Thus, the over-excitation of the NP-contained target using the stronger fluence of the HPs led to the appearance of a large number of ions and free electrons, causing a decrease in the HHG efficiency.

The TCP-induced enhancement of harmonics has previously been demonstrated in both gases and laser-induced plasmas. The first observation of an increase in the harmonic conversion efficiency from plasma plumes irradiated by an intense two-color femtosecond pulse was reported in [27]. A consistent theoretical study of such nonlinear interactions between complex structures (extended plasmas, multijet plasmas, quantum dots, etc.) and multicomponent laser fields constitutes a challenge for modern theoretical approaches. In the course of these studies, it is not enough to solve the problem at the microscopic (atomic) level of interaction [28,29], but it is critical to consider the peculiarities of the laser field propagation in the media. The latter (macroscopic) type of interaction becomes more important in the case of studying complex reactions of the medium.

The efficiency of harmonics produced by a shorter wavelength source became higher due to the strong wavelength-dependent harmonic yield (*I*_harm_ ∞ *λ*^−5^) (*I*_harm_ is the harmonic intensity and *λ* is the driving field wavelength [30]). The *I*_harm_ ∞ *λ*^−5^ rule led to a significant increase in the harmonic yield in the case of the shorter wavelength sources compared to the 806 nm pump. The application of a twice smaller wavelength of driving pulses at other similar conditions should result in a 32-fold growth of harmonic emission. This rule becomes diminished due to the smaller intensity of the second harmonic field. Nevertheless, in many cases, the intensity of even harmonics becomes stronger than that of odd harmonics. There is an explanation stipulating that a strong harmonic generation in the case of TCP is possible due to the formation of a quasi-linear field, the selection of a short quantum path component, which has a denser electron wave packet, and a higher ionization rate compared with the single-color pump [31,32]. With suitable control of the relative phase between the fundamental and the second harmonic radiation, the latter field enhances the short path contribution, resulting in a clean spectrum of harmonics.

**Figure 3:**
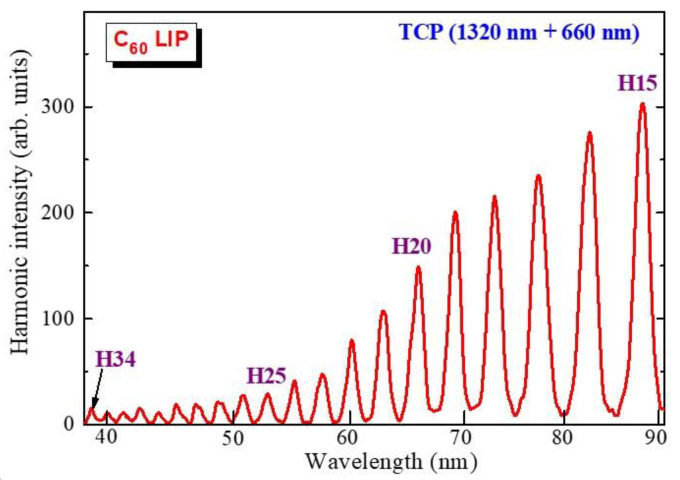
Harmonic spectrum in the case of the two-color pump of fullerene plasma (1320 nm and 660 nm).

Another multiparticle molecule studied was fullerene. This molecule contains 60 carbon atoms and can be considered an attractive medium for HHG [33]. In order to expand the scope of the HHG-related nonlinear spectroscopy of various ablated materials, it was necessary to use tunable femtosecond radiation. Fullerene plasma demonstrated some specific nonlinear optical properties that allowed the odd and even harmonics of the 1320 nm + 660 nm pump to be expanded (Figure 3). In this case, the harmonic cut-off was H34.

The harmonic spectra were measured with a microchannel plate (MCP), and then a CCD camera collected the images of the spectra that appeared on the back side of the MCP (i.e., in the phosphor screen). The data collected by the CCD camera were saved on a PC and then the ImageJ program allowed for determining the intensity distribution of the harmonics along the XUV region (Figure 3). These measurements were carried out in conditions where the collected images represented the unsaturated data. The term “arbitrary units” (arb. units) presented in a few figures describes the relative intensities of the components of the XUV spectra in the case of the analysis of the unsaturated raw images of harmonics distribution. 

### 2.3. Quasi-Phase-Matching of the Group of Harmonics in Molecular Plasmas

**Figure 4:**
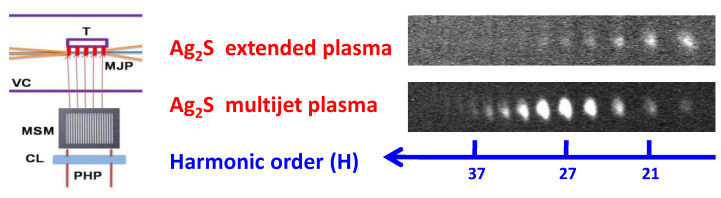
QPM scheme and raw images of harmonic spectra in the case of SCP (806 nm) of silver sulfide LIP. Left panel: experimental setup. VC: vacuum chamber; T: target; MJP: multijet plasma; MSM: multislit mask; CL: cylindrical lens; PHP: picosecond heating pulse. Right panels: raw images of harmonics distribution. Right upper panel: extended homogeneous plasma. Right bottom panel: 5-jet plasma.

Previously, quasi-phase-matching (QPM) was reported in cases of gas jets and atomic plasmas [34,35,36]. In the present study, the application of silver sulfide as the 5 mm long target for ablation allowed for the demonstration of the QPM effect in the molecular plasma. The focusing of heating pulses using a cylindrical lens allowed for achieving the extended plasma. The raw image of the harmonic spectrum from the extended Ag_2_S plasma is presented in the upper panel of Figure 4. The featureless harmonic spectrum up to H31 showed a steady drop in the harmonic yield in the XUV range. The application of the multislit mask (MSM, left panel of Figure 4) placed between the cylindrical lens and target allowed for the formation of the multijet plasma instead of the extended imperforated LIP. The size of the slits was 0.5 mm with a distance of 0.5 mm between them.

The bottom panel of Figure 4 shows the raw spectrum of the harmonics obtained using the insertion of the MSM on the path of the heating beam. One can see a significant variation in the harmonics intensity distribution compared with the case of the imperforated plasma. A group of harmonics (H23 to H33) was enhanced in the 24–35 nm spectral range. The maximum enhancement was achieved for H27.

**Figure 5:**
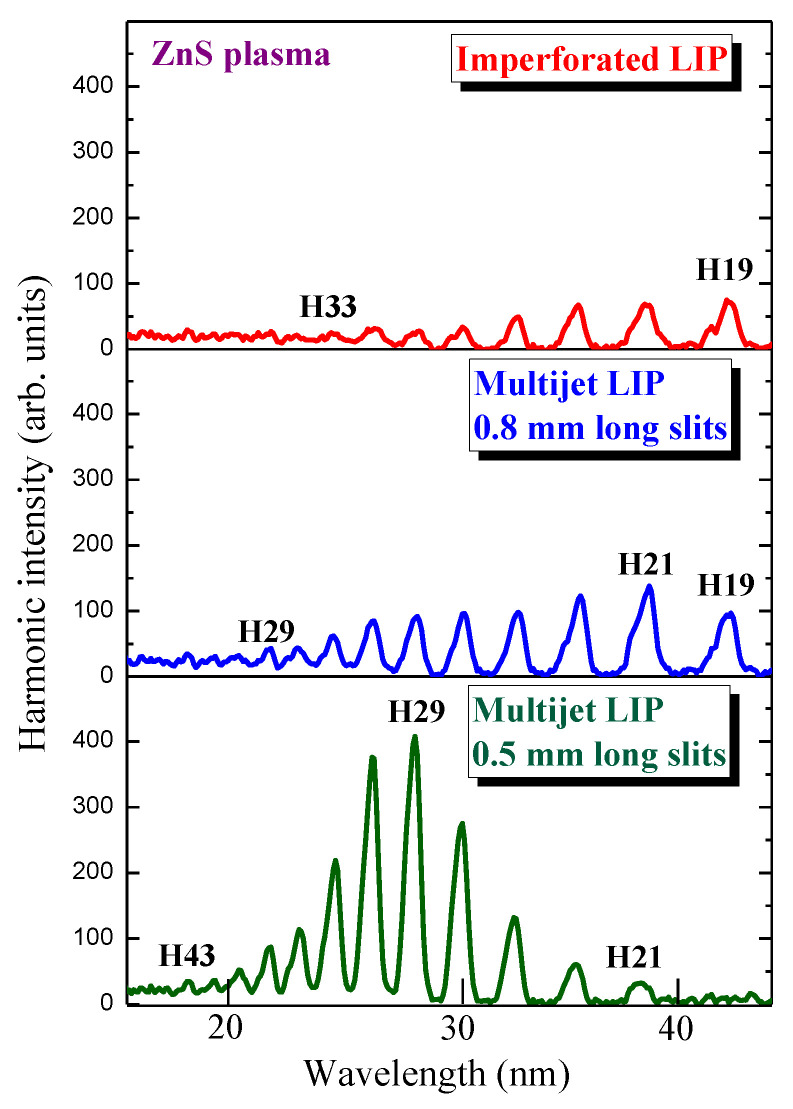
Harmonic intensities in the case of different ZnS plasma configurations. Upper panel: imperforated LIP. Middle panel: multijet plasma using the MSM with the slit size of 0.8 mm. Bottom panel: multijet plasma using the MSM with the slit size of 0.5 mm.

I analyzed the influence of the plasma jet sizes on the enhancement of the groups of the QPM harmonics in the case of the extended ZnS plasma. I changed the MSMs by varying the sizes of the silts from 0.5 to 0.8 mm. As in the case of the silver sulfide plasma, I first analyzed the HHG spectrum using the extended zinc sulfide LIP (upper panel of Figure 5). 

Weak decaying harmonics up to H33 were observed in this experiment. Once I inserted the MSM with the slit size of 0.8 mm, an enhancement, though not as strong as in the case shown in Figure 4, of the lower-order harmonics was observed (middle panel of Figure 5). The replacement of this MSM with the one used in the case of the silver sulfide plasma (i.e., with slit sizes of 0.5 mm) allowed for a significant enhancement of the harmonics centered at H29 (bottom panel of Figure 5).

With these experiments, I showed the realization of the QPM of a group of harmonics generated in multijet plasma produced by a laser ablation of Ag_2_S and ZnS using 806 nm laser pulses and presented a more than 20-fold growth of the shorter wavelength harmonics generated in the multijet molecular LIP compared with the imperforated extended plasma.

## 3. Experimental Arrangements

**Figure 6:**
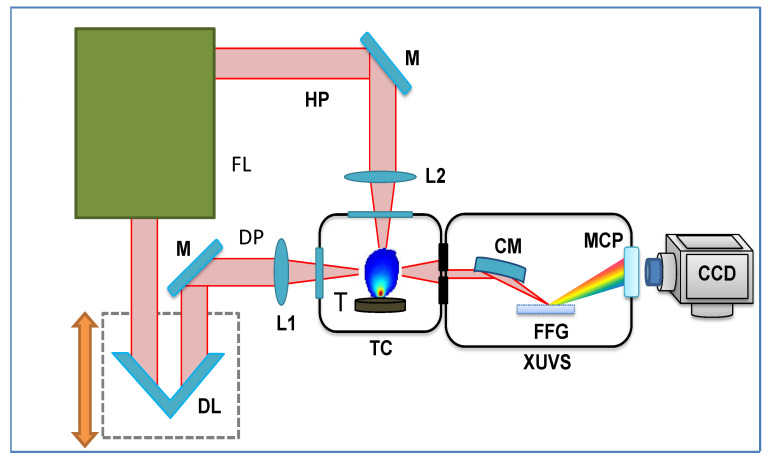
Experimental setup for HHG in molecular plasmas. FL: femtosecond laser (either 806 nm or tunable NIR); M: mirror; L1 and L2: focusing lenses for uncompressed 370 ps heating pulses (HPs) and 65 fs driving pulses (DPs); DL: optical delay line; TC: target chamber; XUVS: extreme ultraviolet spectrometer; T: molecular target; CM: cylindrical mirror; FFG: flat-field grating; MCP: microchannel plate; CCD: camera.

Various molecular structures (Mn_2_O_3_ NPs, In_2_O_3_ NPs, C_60_, Cr_3_C_2_, Ag_2_S and ZnS, all purchased from Sigma-Aldrich) were used in this study. Ti:sapphire laser allowed for the generation of 806 nm, 370 ps of uncompressed pulses at a pulse energy of 6 mJ to heat the molecular targets using a spherical lens with a focal length of 200 mm and form the LIP (Figure 6). SCP (806 nm, 64 fs, 5 × 10^14^ W/cm^2^) and a TCP (806 nm + 403 nm) schemes of laser–plasma interactions were used. The DPs propagated at a distance of ~0.2 mm above the target surface. The delay between the HPs and DPs varied between 60 and 100 ns. During the study, near-infrared radiation (1 mJ, 70 fs, 1280–1440 nm and its second harmonic) was used as well. The high-order harmonics were analyzed with an XUV spectrometer. 

## 4. Conclusions

In conclusion, I demonstrated the optimization of the molecular laser-induced plasma formation from the point of view of a high harmonic yield during the propagation of the femtosecond pulses through different molecular plasma formations. I showed the conditions of molecular target ablation when the resonance-induced processes started to play a decisive role in the enhancement of a single harmonic. The application of a two-color pump for these plasmas allowed for the enhancement of odd orders and the generation of even orders of harmonics. I also showed perspectives on the enhancement of a whole set of harmonics when the femtosecond pulses propagated through the nanoparticles comprising the molecular structures. Finally, I demonstrated the quasi-phase-matching of the group of harmonics generated in the molecular LIP.

## Data Availability

Data underlying the results presented in this paper are not publicly available at this time, but may be obtained from the author upon reasonable request.
